# Supplementary material for: Evaluation of biomechanically corrected intraocular pressure using Corvis ST and comparison of the Corvis ST, noncontact tonometer, and Goldmann applanation tonometer in patients with glaucoma
Source: PLoS One. 2020 Sep 23;15(9):e0238395. doi: 10.1371/journal.pone.0238395 (PMC7510959; doi:10.1371/journal.pone.0238395)
Supplement: Attachment — Submitted filename: Response to Reviewers.docx [file pone.0238395.s001.docx]

PONE-D-20-07468
Evaluation of biomechanically corrected intraocular pressure using Corvis ST and comparison of the Corvis ST, noncontact tonometer, and Goldmann applanation tonometer in patients with glaucoma
PLOS ONE

Dear Dr Nakao,

Thank you for submitting your manuscript to PLOS ONE. After careful consideration, we feel that it has merit but does not fully meet PLOS ONE’s publication criteria as it currently stands. Therefore, we invite you to submit a revised version of the manuscript that addresses the points raised during the review process.


We would appreciate receiving your revised manuscript by Jun 19 2020 11:59PM. When you are ready to submit your revision, log on to https://www.editorialmanager.com/pone/ and select the 'Submissions Needing Revision' folder to locate your manuscript file.

If you would like to make changes to your financial disclosure, please include your updated statement in your cover letter.

To enhance the reproducibility of your results, we recommend that if applicable you deposit your laboratory protocols in protocols.io, where a protocol can be assigned its own identifier (DOI) such that it can be cited independently in the future. For instructions see: <http://journals.plos.org/plosone/s/submission-guidelines#loc-laboratory-protocols>

Please include the following items when submitting your revised manuscript:

- A rebuttal letter that responds to each point raised by the academic editor and reviewer(s). This letter should be uploaded as separate file and labeled 'Response to Reviewers'.
- A marked-up copy of your manuscript that highlights changes made to the original version. This file should be uploaded as separate file and labeled 'Revised Manuscript with Track Changes'.
- An unmarked version of your revised paper without tracked changes. This file should be uploaded as separate file and labeled 'Manuscript'.

Please note while forming your response, if your article is accepted, you may have the opportunity to make the peer review history publicly available. The record will include editor decision letters (with reviews) and your responses to reviewer comments. If eligible, we will contact you to opt in or out.

We look forward to receiving your revised manuscript.

Kind regards,

Rajiv R. Mohan, Ph.D.
Academic Editor
PLOS ONE


Additional Editor Comments (if provided):

Dear authors,
I wish to inform that your MSS has been reviewed by the two reviewers and EBM, and requires addressing of some key points/issues. It is recommended to read the concerns and address them appropriately for the consideration of publication.
Thank you
Rajiv Mohan

Journal requirements:

When submitting your revision, we need you to address these additional requirements:

1.    Please ensure that your manuscript meets PLOS ONE's style requirements, including those for file naming. The PLOS ONE style templates can be found at http://www.plosone.org/attachments/PLOSOne_formatting_sample_main_body.pdf and http://www.plosone.org/attachments/PLOSOne_formatting_sample_title_authors_affiliations.pdf

2. We note that you have stated that you will provide repository information for your data at acceptance. Should your manuscript be accepted for publication, we will hold it until you provide the relevant accession numbers or DOIs necessary to access your data. If you wish to make changes to your Data Availability statement, please describe these changes in your cover letter and we will update your Data Availability statement to reflect the information you provide.

[Note: HTML markup is below. Please do not edit.]

Reviewers' comments:

Reviewer's Responses to Questions

**Comments to the Author**

1. Is the manuscript technically sound, and do the data support the conclusions?

The manuscript must describe a technically sound piece of scientific research with data that supports the conclusions. Experiments must have been conducted rigorously, with appropriate controls, replication, and sample sizes. The conclusions must be drawn appropriately based on the data presented. 

Reviewer #1: Partly

Reviewer #2: Yes

2. Has the statistical analysis been performed appropriately and rigorously? 

Reviewer #1: Yes

Reviewer #2: No

3. Have the authors made all data underlying the findings in their manuscript fully available?

The [PLOS Data policy](http://www.plosone.org/static/policies.action#sharing) requires authors to make all data underlying the findings described in their manuscript fully available without restriction, with rare exception (please refer to the Data Availability Statement in the manuscript PDF file). The data should be provided as part of the manuscript or its supporting information, or deposited to a public repository. For example, in addition to summary statistics, the data points behind means, medians and variance measures should be available. If there are restrictions on publicly sharing data—e.g. participant privacy or use of data from a third party—those must be specified.

Reviewer #1: Yes

Reviewer #2: Yes

4. Is the manuscript presented in an intelligible fashion and written in standard English?

PLOS ONE does not copyedit accepted manuscripts, so the language in submitted articles must be clear, correct, and unambiguous. Any typographical or grammatical errors should be corrected at revision, so please note any specific errors here.

Reviewer #1: Yes

Reviewer #2: Yes

5. Review Comments to the Author

Please use the space provided to explain your answers to the questions above. You may also include additional comments for the author, including concerns about dual publication, research ethics, or publication ethics. (Please upload your review as an attachment if it exceeds 20,000 characters)
Dear Dr Rajiv R. Mohan, we would like to thank you and the reviewers for their constructive and insightful reviews. We believe that the points raised, and our revisions, have resulted in an improved manuscript again. Our responses to the suggested revisions follow (written in red).

Reviewer #1:

Thank you very much. Please find the comments and corrections added in the manuscript.

This is an interesting and current article but needs adjustments such as improving the explanation in the introduction on the thickness and biomechanics of the cornea, citing the competing device (ORA) that was the first to use this type of technology and giving more details on how Corvis works ST.

Thank you very much for this comment. We agree with you. We added the sentence below in the introduction section (Page ４ Line 52).

“Nevertheless, IOP readings increased as the CCT increased [2-12], axial length decreased [13, 14], age increased [9, 12], and the corneal curvature decreased [8, 15] when the two tonometers are used.

In 2005, to provide a corrected IOP unaffected by CCT, the Ocular Response Analyzer (ORA; Reichert, Delpew, NY, USA) was introduced [16]. The ORA was the first non-contact tonometer to convert corneal biomechanics into numerical values using dynamic infrared signal analysis of the corneal biomechanical response. However, the ORA cannot provide a direct description of the mechanical behavior of the cornea.

More recently, the Corneal Visualization Scheimpflug Technology (Corvis ST; Oculus, Wetzlar, Germany) has been introduced as a novel non-contact tonometer designed to accurately measure IOP and the detailed biomechanical response of the cornea to an air pulse. The Corvis ST records the corneal reaction to a defined air pulse with a Scheimpflug imaging system that takes about 4,330 images per second. Then, it estimates the corrected IOP without the influence of ocular biomechanical parameters, including CCT or aging; it is named as IOPpachy-Corvis.”

In the methodology, you should mention inclusion criteria (minimum visual acuity, how many drugs the patient is using, minimum and maximum IOP included in the work), think about including pregnant women and ocular hypertensive patients in the exclusion criteria and better explain the power of the sample.

Thank you very much. We totally agree with you. We did not consider the inclusion criteria about minimum visual acuity, how many drugs the patient is using, minimum and maximum IOP in advance. As discussed above, we added the limitations in the discussion section (Page 18 Line 266).

“Second, our participants included only patients with POAG. It is unclear whether our results can be applicable to healthy patients and those without POAG. Third, our participants were treated using antiglaucoma medications, which are intended to reduce elevated intraocular pressure. Thus, the IOPs measured in our participants all seemed within the normal range. Our results may not generalize to those with higher IOP measurements. Fourth, our study did not consider other factors that may influence IOP readings, such as visual acuity [29, 30] and number of antiglaucoma medications [31, 32], and future research is desired in this aspect.”

And we added the visual acuity data (mean±standard deviation and range) in the table1 (Page 9 Line 142)

Table 1. Demographics and ocular characteristics of patients (POAG, n=71)

|  | Mean ± SD | Range |
| --- | --- | --- |
| Visual acuity (logMAR) | -0.005 ± 0.21 | -1.08 – 1.00 |
| Age (year) | 62.75 ± 11.68 | 30.8 – 79.4 |
| Sex (n, female) | 31 | |
| Axial length (mm) | 25.16 ± 1.74 | 22.1 – 29.2 |
| Average corneal curvature (mm) | 7.73 ± 0.28 | 7.14 – 8.45 |
| Central corneal thickness (μm) | 536 ± 33.85 | 460 – 635 |

POAG, primary open-angle glaucoma; logMAR, logarithm of the minimum angle of resolution; SD, standard deviation

We include a pregnant women and ocular hypertensive patients in the exclusion criteria in the Materials and Methods section (Page7 Line107).

“The exclusion criteria included intraocular surgery or refractive laser treatment and any systemic or ocular pathology that could affect the IOP measurements; refractive error equal to or exceeding -6.00 diopter equivalent sphere, and corneal astigmatism equal to or exceeding 3.00 diopters; ocular hypertension; diabetes [19]; pregnancy [20]; and Scheimpflug images with a low quality, which cannot be automatically analyzed.”

And we tried to explain the power of the sample in the statistical analysis section (Page 8 Line 122/ Page 9 Line 132).

“IOP measurements were compared using a repeated measures ANOVA and a paired t-test with Bonferroni correction. A sample size of 50 participants was needed to achieve 80% power to detect a minimal clinically important difference of 1.5 mmHg, assuming a standard deviation of 1.75 mmHg in the differences between IOPs, a significance level of 0.05, and a 10% patient ineligibility.”

“The target sample size estimates were based on the effect size f2 (0.15); number of predictors (4); significance level (0.05); and power (80%). Considering 10% of patients to be ineligible, the target sample size was determined to be at least 61 patients. "

It uses adequate statistics and correlates well the data of the proposed methodology.
In the discussion, he concludes about the proposed methodology and results and reports the negative points. Among them, there is a fundamental issue that would be the inclusion of a control group to correlate and compare data from patients with glaucoma.

Thank you so much. We totally agree with you. In the future, we need to compare between healthy subjects and patients with glaucoma. We added the sentence below to the limitation in the discussion section (Page 18 Line 266).

“Second, our participants included only patients with POAG. It is unclear whether our results can be applicable to healthy patients and those without POAG.”

Reviewer #2: The paper is written to investigate the IOP measured by Corvis, NCT and GAT. However, there are still some questions existed and need to be checked.

Thank you so much. Please find the comments and corrections added in the manuscript.

Major comments
1. The IOP values in glaucoma is not exactly same to normal eyes, it should add the comparsion of the IOP measured by Corvis, NCT and GAT in normal eyes.

Thank you very much. We totally agree with you and Reviewer1. In the future, we need to compare between healthy subjects and patients with glaucoma. We added the sentence below to the limitation in the discussion section (Page 18 Line 266).

“Second, our participants included only patients with POAG. It is unclear whether our results can be applicable to healthy patients and those without POAG.”

2. The author found the IOP-GAT measurements had the highest values (13.5 ± 2.1 mmHg [mean ± SD]), followed by IOP-NCT (13.2 ± 2.7 mmHg), IOP-Corvis (10.6 ± 2.8 mmHg), and bIOP-Corvis (10.0 ± 2.3 mmHg). The statistically analysis (such as analysis of variance, and SNK method) is needed to compare the IOP values.

Thank you very much. We agree with your opinion. As discussed above, we decided to utilize a repeated measures ANOVA and a paired t-test with Bonferroni correction.

We added the sentence below in the abstract section (Page 2 Line 26)

“We included 71 patients with primary open-angle glaucoma and assessed their IOP measurements obtained with the GAT, NCT, and Corvis ST using a repeated measures ANOVA, a paired t-test with Bonferroni correction, stepwise multiple regression analyses and Bland–Altman plots.”

And in the statistical analysis section (Page 8 Line 122)

“IOP measurements were compared using a repeated measures ANOVA and a paired t-test with Bonferroni correction.”

And in the results section (Page 10 Line 155).

“We found a significant difference between the IOP measurements by using a repeated measures ANOVA (all, p <0.001). In the paired t-test with Bonferroni correction, bIOP-Corvis obtained significantly the lowest values of the four IOP measurements (all, p <0.001). IOP-Corvis produced significantly lower values than IOP-NCT and IOP-GAT (all, p <0.001); however, we found no significant differences between IOP-NCT and IOP-GAT (p >0.05).”

3. In Table 2, the author used the univariate and multivariate regression analyses. There are no significantly differences of bIOP-Corvis and IOP-GAT in the univariate results, which should not further conduct multivariate regression analyses, please checks it.

| **Independent variables** | **IOP measurements** | | | | | | | | | | | |
| --- | --- | --- | --- | --- | --- | --- | --- | --- | --- | --- | --- | --- |
|  | **bIOP-Corvis** | | | **IOP-Corvis** | | | **IOP-NCT** | | | **IOP-GAT** | | |
|  | **Standardized β** | **p** | **VFI** | **Standardized β** | **p** | **VFI** | **Standardized β** | **p** | **VFI** | **Standardized β** | **p** | **VFI** |
| **Univariate regression analysis** | | | | | | | | | | | | |
| Age (year) | -0.13 | 0.282 |  | 0.00 | 0.992 |  | -0.161 | 0.180 |  | 0.004 | 0.976 |  |
| Average corneal curvature (mm) | -0.16 | 0.177 |  | -0.18 | 0.142 |  | 0.076 | 0.527 |  | -0.050 | 0.677 |  |
| Axial length (mm) | 0.05 | 0.697 |  | 0.04 | 0.748 |  | 0.164 | 0.172 |  | 0.030 | 0.802 |  |
| Central corneal thickness (μm) | 0.01 | 0.926 |  | 0.34 | 0.004 |  | 0.419 | 0.000 |  | 0.179 | 0.136 |  |
| **Stepwise multivariate regression analysis** | | | | | | | | | | | | |
| Age (year) |  |  | 1.1 |  |  |  |  |  |  |  |  |  |
| Average corneal curvature (mm) |  |  | 1.1 | -0.19 | 0.089 | 1.0 |  |  |  |  |  |  |
| Axial length (mm) |  |  |  |  |  |  |  |  |  |  |  |  |
| Central corneal thickness (μm) |  |  |  | 0.35 | 0.003 | 1.0 | 0.419 | 0.000 |  |  |  |  |

Thank you so much. We agree with you. We do not need to further conduct multivariate regression analyses for bIOP-Corvis and IOP-GAT. We corrected the table 2 (Page 12)

4. The clinical application of four IOP values needs to be discussed and the discussion should more deeply.

Thank you very much. As discussed above, we added the sentence below in the discussion section (Page 17 Line 251).

“Advantages and disadvantages of bIOP-Corvis

Most commercially available tonometers estimate IOP based on the corneal applanation or indentation. Therefore, measured values of IOP are generally influenced by corneal biomechanics. The bIOP-Corvis is an estimate of the corrected IOP, which is minimally influenced by ocular biomechanical parameters. In this study, we used regression analyses to investigate the anatomical and structural factors that affect the bIOP-Corvis measurements. We found that age, average corneal curvature, axial length, and CCT were not significant factors that influence bIOP-Corvis. However, our result of the Bland–Altman plots for bIOP-Corvis and IOP-GAT showed that fixed biases were identified. This means that bIOP-Corvis significantly underestimated IOP using the standard clinical tonometer for measurements. Nevertheless, proportional bias was not between the bIOP-Corvis and IOP-GAT measurements and the 95% LOAs was relatively low. Therefore, it seems that we can convert from bIOP-Corvis to IOP-GAT. Our result implied that the bIOP-Corvis is a useful IOP value in patients with POAG.”

Minor comments.
1. Page 14 line 12, check the sentence “This showed that that the……”

Thank you so much. We corrected the sentence (Page 16 Line 236).

“This showed that the differences between two IOP values (bIOP-Corvis and IOP-GAT) neither increase nor decrease in proportion to the average values; therefore, it can be easily converted from bIOP-Corvis to IOP-GAT.”

2. the Corvis ST only occurred in title, which is not mentioned in main documents.

Thank you very much. We added the sentence below in the abstract section (Page 2 Line 19)

“The aim of the study was to investigate the effects of various anatomical structures on intraocular pressure (IOP) measurements obtained by the Corneal Visualization Scheimpflug Technology (Corvis ST)”

And in the introduction section (Page 5 Line 64).

“More recently, the Corneal Visualization Scheimpflug Technology (Corvis ST; Oculus, Wetzlar, Germany) has been introduced as a novel non-contact tonometer designed to accurately measure IOP and the detailed biomechanical response of the cornea to an air pulse. The Corvis ST records the corneal reaction to a defined air pulse with a Scheimpflug imaging system that takes about 4,330 images per second.”

And we added the words ”Corvis ST” below in Page 5 Line73/ Page6 Line 91/ Page 7 Line 112/ Page 8 Line 114, 117/ Page 10 Line 152/ Page 13 Line 179, 182/ Page 17 Line 249/ Page 18 Line 265/ Page 19 Line 285

6. PLOS authors have the option to publish the peer review history of their article ([what does this mean?](https://journals.plos.org/plosone/s/editorial-and-peer-review-process#loc-peer-review-history)). If published, this will include your full peer review and any attached files.


If you choose “no”, your identity will remain anonymous but your review may still be made public.


**Do you want your identity to be public for this peer review?** For information about this choice, including consent withdrawal, please see our [Privacy Policy](https://www.plos.org/privacy-policy).

Reviewer #1: No

Reviewer #2: No


[NOTE: If reviewer comments were submitted as an attachment file, they will be attached to this email and accessible via the submission site. Please log into your account, locate the manuscript record, and check for the action link "View Attachments". If this link does not appear, there are no attachment files to be viewed.]


While revising your submission, please upload your figure files to the Preflight Analysis and Conversion Engine (PACE) digital diagnostic tool, <https://pacev2.apexcovantage.com/>. PACE helps ensure that figures meet PLOS requirements. To use PACE, you must first register as a user. Registration is free. Then, login and navigate to the UPLOAD tab, where you will find detailed instructions on how to use the tool. If you encounter any issues or have any questions when using PACE, please email us at [figures@plos.org](mailto:figures@plos.org). Please note that Supporting Information files do not need this step.

*In compliance with data protection regulations, you may request that we remove your personal registration details at any time.*  [*(Remove my information/details)*](https://www.editorialmanager.com/pone/login.asp?a=r)*. Please contact the publication office if you have any questions.*
